# Rosin from *Pinus pinaster* Portuguese forests shows a regular profile of resin acids

**DOI:** 10.3389/fpls.2023.1268887

**Published:** 2023-10-26

**Authors:** Ângela Pinheiro, Isabel Martins, Artur Bento, Rita Escórcio, Carolina Nunes, Adélia Varela, João Nunes, Carlos A.M. Afonso, Cristina Silva Pereira

**Affiliations:** ^1^ Instituto de Tecnologia Química e Biológica António Xavier, Universidade Nova de Lisboa (ITQB NOVA), Oeiras, Portugal; ^2^ Association BLC3 – Technology and Innovation Campus, Centre Bio R&D Unit, Rua Nossa Senhora da Conceição n2, Oliveira do Hospital, Portugal; ^3^ Instituto Nacional Investigacão Agrária e Veterinária, Oeiras, Portugal; ^4^ Research Institute for Medicines (iMed.ULisboa), Faculty of Pharmacy, Universidade de Lisboa, Lisboa, Portugal

**Keywords:** abietic acid, dehydroabietic acid, *Pinus pinaster* oleoresin, rosin, nuclear magnetic resonance method, resin acids

## Abstract

*Pinus pinaster* forestry occupies >20% of the forest ecosystem area in the continental territory of Portugal with a high impact on the national economy. This species’ major derived non-wood product is oleoresin, the raw material for rosin production. Rosin comprises mainly a blend of resin acids and has broad industrial and pharmaceutical applications. Oleoresin production in Portugal has been progressively reduced due to low-cost producers in other countries; currently, it reaches only 2% of the existing *P. pinaster* trees. To support this value chain, the chemical fingerprint of rosin derived from the national forest requires focused analysis. In the present study, we collected oleoresin within seven geographically distinct pure *P. pinaster* forests in two consecutive collection years. A high-resolution nuclear magnetic resonance (NMR) method was used to quantify the diversity of resin acids in the corresponding rosin samples. Overall, the acquired data highlighted that the profile of resin acids in *P. pinaster* rosin produced in Portugal is highly regular, regardless of the forest location, having as the major constituents abietic acid and dehydroabietic acid. The diversity of resin acids is possibly influenced, to a minor extent, by some edaphoclimatic factors.

## 1 Introduction

In Portugal, forest occupies up to 36% (3.2 million ha) of the national territory, and it is the main land use, representing one of the most important bioeconomy value chains. Currently, the Portuguese pine forest ecosystem covers nearly 1 million ha and is dominated by maritime pine (*Pinus pinaster* Aiton) and stone pine (*Pinus pinea* L.), representing 22% and 6% of the total forest area, respectively (ICNF, 2015). *P. pinaster* is the most relevant silvicultural coniferous tree in Portugal, primarily exploited for the production of by-products of wood, paper, and oleoresin (often defined simply as resin) (Reboredo, 2014; Sousa et al., 2018; Neis et al., 2019). However, this species has shown a decrease in occupied forest areas deeply affected by fires and pests (ICNF, 2015; Mantas et al., 2022). Until 1980, Portugal was an important producer and exporter of oleoresin with production yields above 100,000 tons/year. However, since then, a progressive decline in the Portuguese resin tapping activity has occurred due to competition from petroleum-based counterparts and production at lower costs in other producing countries, e.g., China and Brazil (López-Álvarez et al., 2023). Between 2018 and 2019, only 2% of the *P. pinaster* and 1% of the *P. pinea* trees were explored, producing *ca.* 6,310 tons/year of resin (ICNF, 2015; CentroPinus, 2020). The rise in the cost of fossil resources globally is anticipated to stimulate oleoresin exploitation, e.g., designing new chemicals and materials (López-Álvarez et al., 2023).

**Figure 1:**
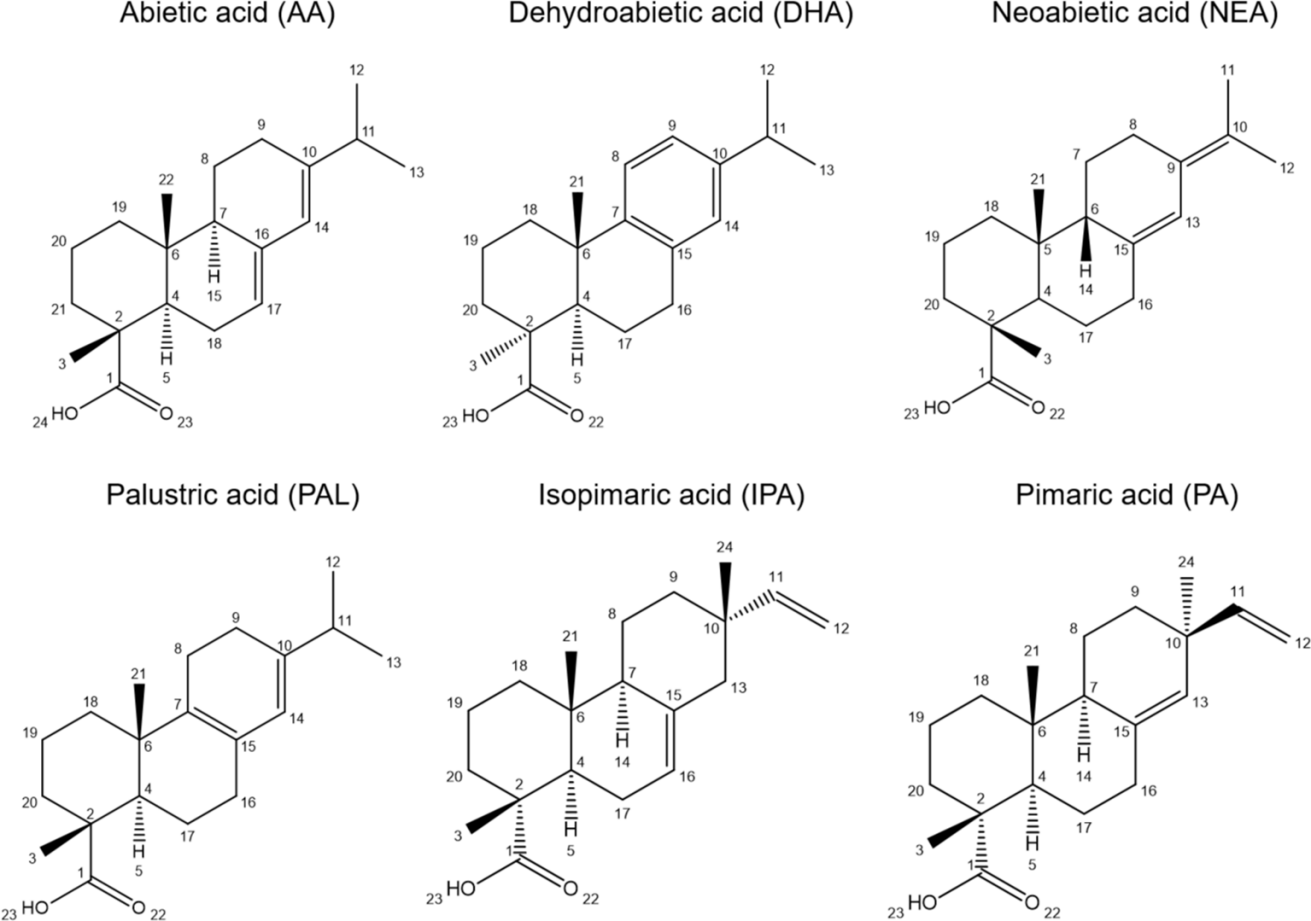
The chemical structures of the diterpenic resin acids from *Pinus pinaster* (and *Pinus pinea*) rosin identified in the present work.

Rosin, which is obtained by steam distillation of oleoresin, as the non-distilled fraction, composing ~95% of its weight, constitutes a sustainable industrial raw material for the production of, e.g., varnishes, chewing gum, emulsifiers, polymers, and coatings (Silvestre and Gandini, 2008; Sacripante et al., 2015; Neis et al., 2019). It is also recognized as a source of bioactive molecules (Celedon and Bohlmann, 2019) with proposed applications in pharmaceuticals, biocides, insect repellents, and antioxidants (Sousa et al., 2018), all constituting promising exploitation alternatives to leverage the Portuguese resin market. Rosin is essentially a blend of resin acids (RAc): diterpenes, which harbor 20 carbons distributed along a six-carbon tricyclic ring, a single carboxylic acid group, and up to three double bonds, which can differ in relative position in the molecule (Neis et al., 2019; Sarria-Villa et al., 2021). Most resin acids composing *P. pinaster* rosin have abietane (acids: abietic, AA; neoabietic, NEA; dehydroabietic, DHA; palustric, PAL; levopimaric, LEV) or pimarane skeletons (acids: pimaric, PA; isopimaric, IPA; sandaracopimaric, SAN) (**Figure 1**). The ratio of resin acids greatly determines the physicochemical properties of oleoresin/rosin, e.g., odor and color, which impact downstream applications, hence the resin value chain. Furthermore, due to the higher susceptibility to chemical alteration, abietanes have a greater potential for biotechnological uses (e.g., production of bioactive compounds) (Silvestre and Gandini, 2008; Kugler et al., 2019).

Increasing the exploitation of Portuguese rosin is challenging due to the multifactorial nature of the problem, including deterioration of forest areas due to fires and pests and erratic forest management practices implemented by a majority of small private owners (~90%, owing in average <0.5 ha) (Reboredo, 2014; CentroPinus, 2020). In addition, the impact of forest location on rosin chemistry constitutes an unresolved question; studies focusing on rosin collected across the national forest are lacking. In the present study, a nuclear magnetic resonance (NMR)-based protocol was used for a swift quantitative analysis of the major resin acids in pine rosin. Data integration builds a bird’s-eye view of the rosin chemistry derived from Portuguese *P. pinaster* forests covering most of the ecoregions of the Iberian Peninsula. Finally, the correlation of rosin chemistry with key edaphoclimatic parameters was verified and discussed in detail.

## 2 Materials and methods

### 2.1 Chemicals

Chloroform (p.a., >99.98%) was purchased from Fisher Chemical (Waltham, MA, USA). Hexadecane (p.a., >99%), 2.0 M of (trimethylsilyl)diazomethane in hexane, *N*,*O*-bis(trimethylsilyl)trifluoroacetamide (p.a., >99%) containing 1% (v/v) of trimethylchlorosilane (BSTFA : TMCS, 99:1), pyridine (p.a., >99%), and 1,4-dioxane (p.a., >98%) were supplied by Sigma-Aldrich (Darmstadt, Germany). Deuterated dimethyl sulfoxide (DMSO-*d*_6_; p.a., >99.99%) was obtained from Merck (Darmstadt, Germany). Resin acid standards, namely, AA (p.a., ≥85%), DHA (p.a., >95%), and IPA (p.a., ≥98%) were purchased from Acros Organics (Verona, Italy), Fluorochem (Hadfield, UK), and Sigma-Aldrich, respectively. All chemicals were of high purity and used without purification.

### 2.2 *Pinus pinaster* resin sampling

**Figure 2:**
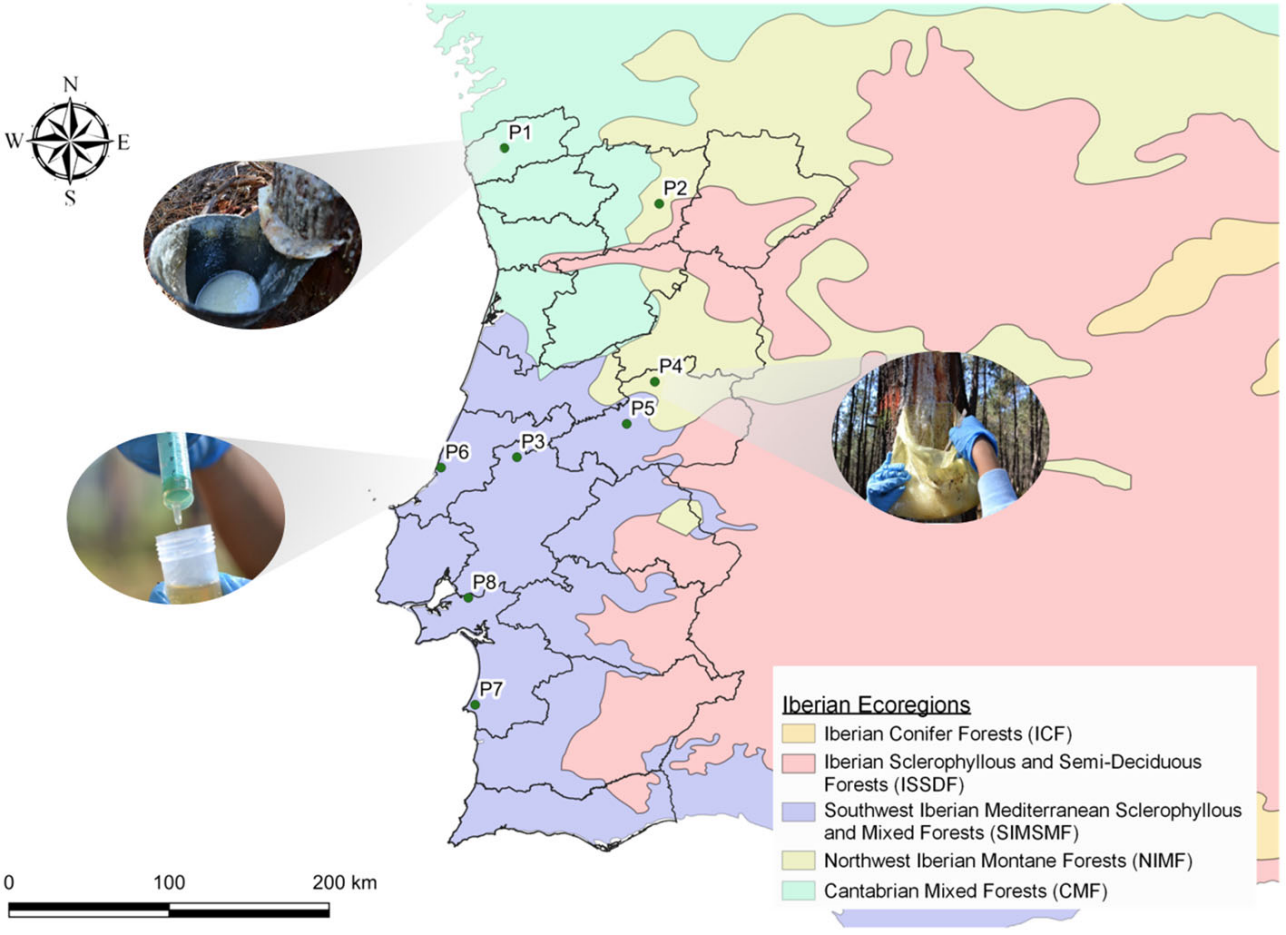
Geographical location of the sampling stands on *Pinus pinaster* Portuguese national forestry, according to Iberian ecoregions (P1–P7). Photographic details on the containers installed for oleoresin tapping (location, P1 and P4) and the sampling method used (P6), are depicted, as described in the “Materials and methods”. Location P8 is a *Pinus pinea* forest. See **Table 1** and **Supplementary Table 1** for further details. Illustration generated using QGIS Development Team (version 3.16), QGIS Geographic Information System, Open Source Geospatial Foundation Project (http://qgis.osgeo.org).

Seven sampling *P. pinaster* pure forests in Portugal were selected (P1 to P7), covering most of the *P. pinaster* forestry that is widely distributed across the country (mainly in the North and Central regions (Reboredo, 2014)) and spread in the three different Iberian ecoregions where *P. pinaster* grows as depicted in **Figure 2** (**Table 1**, **Supplementary Table 1**). Oleoresin collection in each sampling site was carried out three times during the tapping season in Portugal, from July to November 2018: during the summer (two sampling campaigns, July 23 to August 23 and September 18–27) and the autumn (one sampling campaign, October 8 to November 11). New resin samples were collected at some of the sampling sites in September 2019 (P3, P5, P6, and P7): during the summer (one sampling campaign, September 7 to 21) (**Supplementary Table 1**). At each sampling site, a minimum of 20- to 50- ha pine forest was selected. The central hectare was georeferenced and divided into approximately 50 equal squares. The trees in each square were marked with spray paint, and the central pine tree was selected for oleoresin sampling. At P6, due to the smaller forest area, the sampling area had a diameter of 120 m, and 12 trees were sampled per quadrant, plus two trees randomly. To extract oleoresin, a wound of 12 cm maximum width was performed with a chisel for bark chipping at a height of *ca*. 130 cm above the ground, followed in all cases by the application of a stimulating paste containing sulfuric acid. The oleoresin was already being extracted from all the selected trees. Accordingly, the oleoresin samples were collected from the containers (metal or plastic) previously installed. The resin was energetically mixed inside the container with a metal spatula, and *ca.* 10 g was sampled using a plastic syringe (**Figure 2**). The 50 individual oleoresin samples per pine forest were pooled to generate a composite sample representative of the larger population (500 g). In total, 350 trees were sampled per sampling date. Two additional oleoresin samples were collected from *P. pinea* trees (P8) in 2018 during the summer and in 2019 during the winter (**Supplementary Table 1**), which were used as outliers, whenever significant. The samples were placed inside a falcon tube and immediately conserved (dark, 4°C), ensuring maximum preservation until further processing and analysis; all samples were processed simultaneously.

**Table 1:** Specific coordinates of the sampling sites on *Pinus pinaster* Portuguese forestry (provenance codes P1–P7; P8 is a *Pinus pinea* forest).

Species	P. code^(1)^	Province	Municipality	Location	Latitude	Longitude	Elevation (m)	Sea distance (km)	Mean DBH^(2)^ (cm)	Sampling repeats^(3)^
*P. pinaster*	P1	Viana do Castelo	Paredes de Coura	Agualonga	41°51′51.5″N	8°36′09.4″W	415	29.894	104	3
	P2	Vila Real	Vila Pouca de Aguiar	Tresminas	41°28′34.2″N	7°31′22.8″W	773	139.968	76	3
	P3	Santarém	Ourém	Caxarias	39°42′35.9″N	8°30′53.4″W	166	59.593	99	4
	P4	Castelo Branco	Covilhã	Tortosendo	40°14′08.3″N	7°33′13.1″W	696	147.391	100	3
	P5	Castelo Branco	Oleiros	Sarnadas S. Simão	39°56′30.9″N	7°44′58.4″W	584	132.551	115	4
	P6	Leiria	Alcobaça	Pataias	39°38′15.0″N	9°02′39.1″W	128	3.676	114	4
	P7	Setúbal	Sines	Sines	37°59′02.5″N	8°48′22.8″W	53	5.849	104	4
*P. pinea*	P8	Setúbal	Alcochete	Alcochete	38°43′47.5″N	8°51′14.6″W	17	0.0318	–	2

(1) P. code stands for provenance code, identifying a set of samples according to sampling location. (2) Mean DHB stands for the mean diameter breast height (cm) of the sampled trees. (3) Sampling repeats indicate the number of sampling replicates performed at each location. The dates of the independent sampling campaigns are depicted in ESI **Supplementary Table 1**. QGis software was used to extract data for each location and **Figure 2**.

### 2.3 Distillation procedure: from resin to rosin

The oleoresin samples were submitted to a steam-distillation process at 130°C, below the oleoresin degradation temperature (Sarria-Villa et al., 2021). This process renders an insoluble residue, rosin (the volatile fraction consists of turpentine). The still-hot rosin was filtered with a 1-mm pore size mesh and conserved in the dark, inside closed containers at room temperature.

### 2.4 Cryogenic grinding process

A RESTCH cryomill equipped with a 5-mL grinding jar with two aluminum grinding balls (5 mm) was used. To homogenize the rosin samples, they were cryogenically milled at −196°C (liquid nitrogen), as follows: 3 min of precooling followed by three milling cycles, each comprising 3 min of milling at 30 Hz plus 0.5 min of intermediate cooling at 5 Hz. The compositional RAc profile of each sample was immediately analyzed (see below).

### 2.5 Nuclear magnetic resonance analyses

To reveal the diversity of resin acids found in the rosin, the cryomilled samples were immediately solubilized in DMSO-*d*_6_ and analyzed without any further treatment or extraction using NMR with an Avance III 800 CRYO (Bruker Biospin, Rheinstetten, Germany). All samples were analyzed in triplicate. All NMR spectra (^1^H, ^1^H–^1^H correlated spectroscopy (COSY), ^1^H–^13^C heteronuclear single quantum coherence (HSQC), and ^1^H–^13^C heteronuclear multiple bond correlation (HMBC)) were acquired in DMSO-*d*_6_ using 5-mm-diameter NMR tubes at 25° C, as follows: 15 mg of cryomilled rosin directly solubilized in 400 µL of DMSO-*d*_6_ with 10 µL of 1,4-dioxane as internal standard. MestReNova, version 11.04-18998 (Mestrelab Research, S.L., Santiago de Compostela, Spain), was used to process the raw data acquired in the Bruker spectrometer. For quantitative ^1^H NMR, 30° radio frequency pulses of 8.1 µs, relaxation delay of 1 s, acquisition time of 2.04 s, and spectral resolution of 0.245 Hz were used. For experimental validation, pure standards of AA, DHA, and IPA were analyzed in parallel (5 mg/400 µL of DMSO-*d*_6_).

### 2.6 Gas chromatography– mass spectrometry analyses

The gas chromatography–mass spectrometry (GC-MS) method selected for resin acid analysis was previously described for bark samples (Bento et al., 2022). Briefly, commercially available standards of AA, DHA, and IPA (alone or mixed) were solubilized in 1 mL of chloroform, and hexadecane was added as the internal standard. The following were added to the solution and vortexed: 50 µL of pyridine and 100 µL BSTFA : TMCS (99:1). Derivatization was allowed to occur for 1 h at 70°C. The derivatives were then analyzed by GC-MS (Agilent (Santa Clara, CA, USA): 7820 A GC and 5977B quadrupole MS; HP-5MS column) operated as follows: 60°C, 6°C/min until 300° C; 300° C during 10 min. Data were acquired using MSD ChemStation (Agilent); compounds were identified based on electron ionization–mass spectrometry (EI-MS) fragmentation patterns and included in the Wiley-NIST reference library. An external quantification method was used, and all standards (alone or mixed) were analyzed in triplicate.

### 2.7 Statistical analyses

Principal component analysis (PCA) of ^1^H NMR spectra of the rosin was performed using a statistical analysis based on RStudio version 1.4.1106 with the Rnmr1D package (Jacob et al., 2017; Jacob, 2018). All spectra (samples analyzed immediately after cryomilling) were first aligned, baseline correction was applied, and the segments [(5.0;4.9), (3.7;3.1), and (2.6;2.35) ppm], which are regions associated with solvents, were deleted. Statistical analyses were applied to test the hypothesis that there are significant differences in the mean concentrations of the resin acids quantified by NMR in the rosin samples associated with different sampling locations. The normality of group means and the homoscedasticity of variances among means were confirmed using the Shapiro–Wilk test and Bartlett’s test, respectively. Non-parametric tests were performed to access distribution differences of the RAc profiles of rosin samples between the sampling sites (Kruskal–Wallis) and sampling campaigns (Wilcoxon). The statistical analysis was based on RStudio version 1.4.1106 with the Coin package (Kuhn, 2008). Differences were considered significant at the *p* < 0.05 level. The relative influence of edaphoclimatic parameters (e.g., % of sand in the soil, elevation, annual precipitation, and annual temperature range) on the resin acid profiles was tested using RStudio version 1.4.1106 with the Vegan package (Dixon, 2003).

The edaphoclimatic parameters used for the canonical correspondence analysis (CCA) were found in publicly available datasets. The climatic data for each location were obtained from the WorldClim v2 set of global bioclimatic variables (Fick and Hijmans, 2017), comprising an average of 30-year climatic data series (defined as matrix A, **Supplementary Table 2**) (WorldClim, 2020). The soil data for the different locations were retrieved from the INFOSOLO database (https://projects.iniav.pt/infosolo/) (defined as matrix B, **Supplementary Table 3**) (INIAV, 2016; Ramos et al., 2017). The climatic parameters measured in the month prior to the sampling were obtained from the IPMA database (https://www.ipma.pt/pt/index.html, defined as matrix C, **Supplementary Table 4**) (IPMA, 2020). Matrix A contains 19 climatic parameters, matrix B contains five edaphic parameters, and matrix C includes six parameters. To reduce the matrices’ complexity, the parameter dependence was tested using PCA. Analysis was performed using RStudio version 1.4.1106 with the Vegan package (Dixon, 2003).

## 3 Results

### 3.1 NMR spectroscopy reveals comparable spectral profiles for all *P. pinaster* rosin composite samples

Representative *P. pinaster* trees were sampled from seven different forests (P1 to P7) in the continental territory of Portugal (**Table 1**). All the sampled trees had no apparent phytosanitary lesions and showed a mean oleoresin (resin) productivity of 2 to 3 kg/tree/year, with some trees reaching up to 4 to 5 kg/tree/year. The tree diameter breast height (DBH; measured at approximately 115 cm above ground level) ranged from 76 to 115 cm (**Table 1**).

Initial reports showed that the profiles of resin acids, i.e., diversity and amount, are affected by the tree growth rate, age, and even site of sample collection (Beltran et al., 2017; Salomé-Abarca et al., 2018; Rubini et al., 2022). In this study, the oleoresin samples for each location were pooled, as usually applied for harvesting samples for industry, rendering location representative samples (**Supplementary Table 1**). All composite oleoresin samples were subjected to steam distillation to obtain the corresponding composite rosin samples, similar to oleoresin industrial processing.

GC-MS was first used for the analysis of resin diterpenic acids in 1957 and has been considered a standard technique since then (Joye and Lawrence, 1967). However, it presents several challenges for RAc analysis, in part due to their structural similarity that hinders separation (Rezzi et al., 2002; Graça et al., 2017; Salomé-Abarca et al., 2018). Aiming for RAc quantification in rosin GC-MS was initially tested. Pure standard compounds AA, DHA, and IPA (commercially available) were used to construct calibration curves for a defined concentration range. Mixtures of the standard compounds in known concentrations were quantified (**Supplementary Figures 1A–C**). The results show that under the conditions used herein, the GC-MS quantification was inaccurate whenever the compounds were analyzed as a mixture (**Supplementary Figure 1D**, **Supplementary Table 5**). The deviation was particularly obvious for the AA and DHA, both compounds having an abietane skeleton. A possible explanation is that these compounds underwent thermal degradation or isomerization at the elevated temperature used for the GC analysis, as previously suggested (Graça et al., 2017; Li et al., 2019). Based on this result, NMR spectroscopy was chosen as the quantification method since it has previously been reported to allow a precise assignment and quantification of the different resin acids (Graça et al., 2017; Ioannidis et al., 2019; Bento et al., 2022).

**Figure 3:**
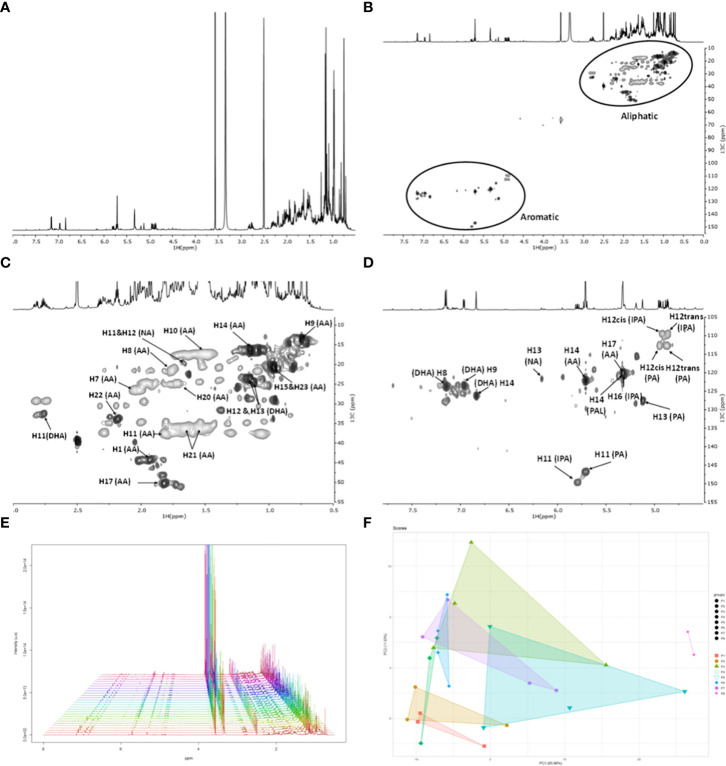
NMR spectral characterization of a rosin sample from *Pinus pinaster*. One sample was randomly selected (first collection date, 2018 from the location of P3). **(A)** The ^1^H NMR and the **(B)** HSQC spectrum: full range and regions corresponding to **(C)** aliphatics and **(D)** aromatics. Some correlations (unlabeled) are uncertain or unidentified. **(E)** ^1^H NMR spectra collected from all the rosin samples (spectra were aligned based on the DMSO peak) and the **(F)** resultant principal component analysis (PCA) (Rnmr1D package). The PCA loadings based on correlation analysis are shown in **Supplementary Figure 8**. NMR, nuclear magnetic resonance; HSQC, heteronuclear single quantum coherence.

To test the NMR method adequacy, a rosin sample was randomly selected (first collection date in 2018 from the location of P3) of which the ^1^H spectrum (**Figure 3A**) and the full-range HSQC spectrum (**Figure 3B**) are shown, highlighting the regions corresponding to aliphatics (**Figure 3C**) and aromatics (**Figure 3D**). The observed ^1^H and ^13^C chemical shifts were assigned using a combination of two-dimensional correlation NMR experiments (^1^H–^1^H: COSY; ^1^H–^13^C: HSQC and HMBC) and previous NMR data on resin acids (Sugimoto et al., 2006; Graça et al., 2017; Ioannidis et al., 2019; Bento et al., 2022). Additional spectra were collected for standards of AA, DHA, and IPA, which further secured the assignments performed (data not shown). On the basis of the HSQC spectrum, the ^1^H chemical shifts of each resin acid could be accurately assigned: AA, DHA, NEA, PAL, IPA, and PA acids (**Figure 1**, **Supplementary Table 6**).

Based on this result, all rosin samples were analyzed by NMR as described above (triplicates). The acquired ^1^H NMR spectra (all presented in high spectral resolution) revealed that the chemical diversity of all the *P. pinaster* rosin samples is qualitatively similar (**Figure 3E**), as also depicted in the 2D correlation NMR experiments (representative spectral example in **Supplementary Figures 2**-**4**; first collection date in 2018 from P4 location). The exception was the P8 samples, corresponding to *P. pinea* rosin, since the corresponding ^1^H NMR spectra did not detect NEA (present in all *P. pinaster* samples) and detected AA at a very low signal intensity compared to the remaining *P. pinaster* rosin samples (**Supplementary Figures 5**-**7**).

PCA reveals that P3, P5, and P7 rosin composite samples have higher dispersion patterns between replicas (**Figure 3F**). The P8 rosin is the most distinct sample. PCA loadings using correlation analysis did not detect any specific NMR spectral signal that would explain any degree of dissimilarity between the rosin samples (**Supplementary Figure 8**). The NMR fingerprints of *P. pinaster* rosin samples made visible their high similarity at a qualitative level. Therefore, to further differentiate their chemical profiles, the NMR quantification of each resin acid was undertaken.

### 3.2 The resin acid profiles of *P. pinaster* rosin composite samples differ at each forest location when different sampling dates are compared

**Figure 4:**
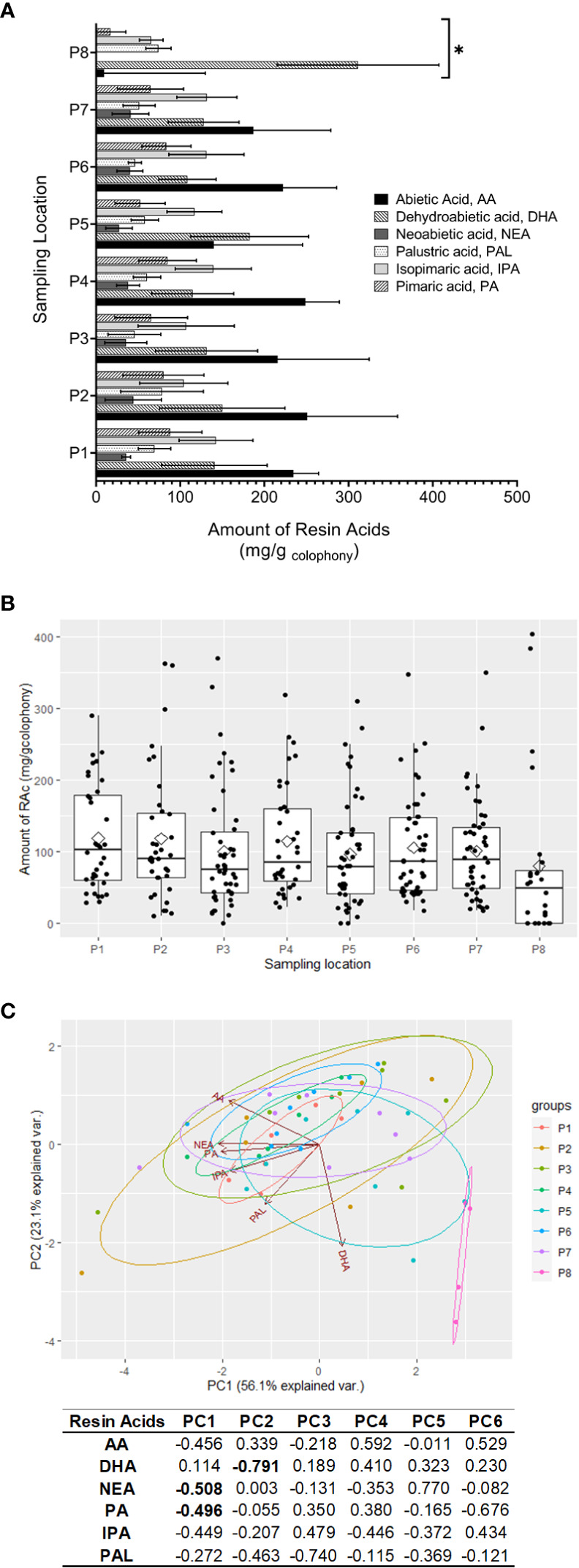
NMR-based quantification of extant resin acids detected in rosin samples of *Pinus pinaster* and *Pinus pinea* (outlier sample). **(A)** The mean abundance (mg/g_rosin_) of the resin acids depicted according to sample (includes mean resin acid amounts and standard deviation bars per location). Resin acids showing statistically relevant differences (P8 location) among samples are highlighted (*Wilcoxon test, *p* < 0.05). **(B)** The mean resin acid amounts and the respective dispersion measures for each quantified resin acid. In the box plots, the boundary of the box closest to zero and farthest from zero indicate the 25^th^ and 75^th^ percentiles, respectively; a black line within the box marks the median. Whiskers above and below the box indicate the 10^th^ and 90^th^ percentiles, respectively. **(C)** Principal component analysis of data in a biplot. The biplot shows sample locations (scores) as labeled dots and the RAc amounts (loadings) as vectors. Circles are for illustrative purposes only. For details on samples, see **Figure 2**, **Table 1**, **Supplementary Table 1**; details on resin acids are in **Figure 1**.

The ^1^H NMR spectra were used to quantify the resin acids in the rosin composite samples (triplicates). Non-overlapping, undoubtedly defined signals of the olefinic, aromatic, and aliphatic protons, were selected for integration, as follows: AA (H-22 at 0.75 ppm), NEA (H-13 at 6.16 ppm), DHA (H-14 at 6.84 ppm), IPA (H-11 at 5.79 ppm), and PA (H-13 at 5.12 ppm) (**Supplementary Figure 9**). The quantification of PAL was inferred by subtracting from the integration of the signal at 5.33 ppm (H-14), which is assigned to both AA and PAL, and the integration of the signal assigned specifically to AA (H-22 at 0.75 ppm). To calculate the amount of each resin acid (mg/g of rosin), the integration values inferred for each resin acid were normalized using the signal integration of the internal standard. **Figure 4A** summarizes the obtained results. **Figure 4B** depicts the box plots of the six quantified resin acids in all rosin samples per sampling location, denoting the samples’ similarity, except those from *P. pinea* that contain DHA as the major RAc. The observed chemical profiles of *P. pinaster* rosin have a prevalent resin acid AA, closely followed by DHA and IPA.

PCA of the variability of the resin acid profiles explained ~56% and ~23% of the total variance by the PC1 and PC2, respectively (**Figure 4C**). All *P. pinaster* rosin composite samples are dissociated from the *P. pinea* rosin, consistent with the PCA of the NMR data (**Figure 3F**). The resin acids explaining the observed differentiation are NEA and PA in PC1 and DHA in PC2. Pairwise Wilcoxon tests further show that only *P. pinea* samples were statistically different.

**Figure 5:**
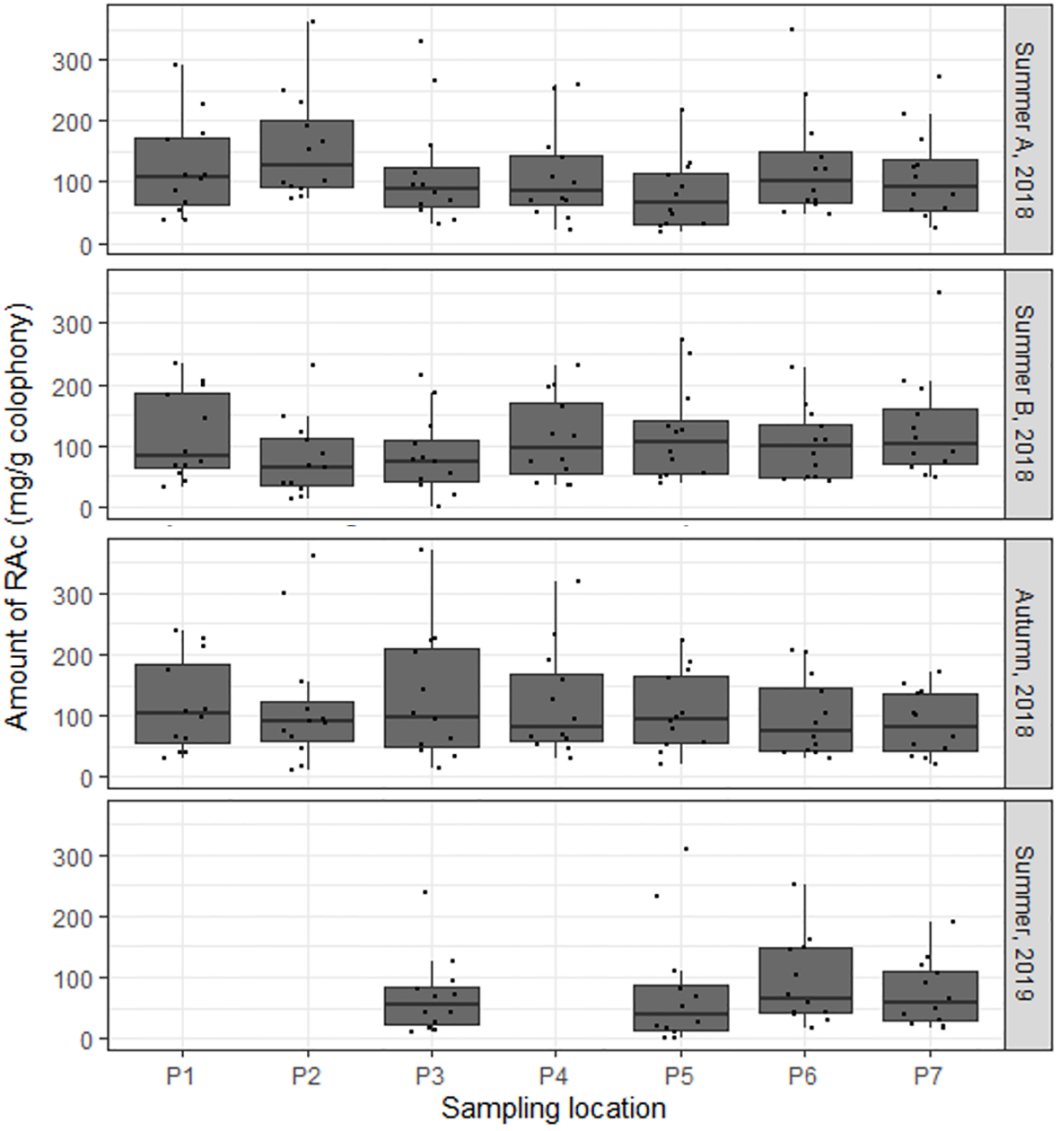
The mean resin acid amounts in Portuguese *Pinus pinaster* rosin samples and respective dispersion measures per sample along time. In the box plots, the boundary of the box closest to zero and farthest from zero indicate the 25^th^ and 75^th^ percentiles, respectively; a black line within the box marks the median. Whiskers above and below the box indicate the 10^th^ and 90^th^ percentiles, respectively. For further details on sampling campaigns, see **Supplementary Table 1**.

The observed uniformity in the RAc profiles of all *P. pinaster* rosin composite samples is due to the high dataset variability where the dates of the sampling campaigns are not differentiated. **Figure 5** depicts the box plots of the *P. pinaster* RAc profiles per sampling location and sampling date (season and year). Comparing the RAc profiles per location along time led to the identification of variations (pairwise Wilcoxon test, *p* < 0.05). It is therefore reasonable to hypothesize that some of the noticed differences might be correlated to climatic factors or other associated edaphic parameters.

### 3.3 The resin acid profiles of *P. pinaster* rosin composite samples show weak correlation with edaphic and climatic parameters

**Figure 6:**
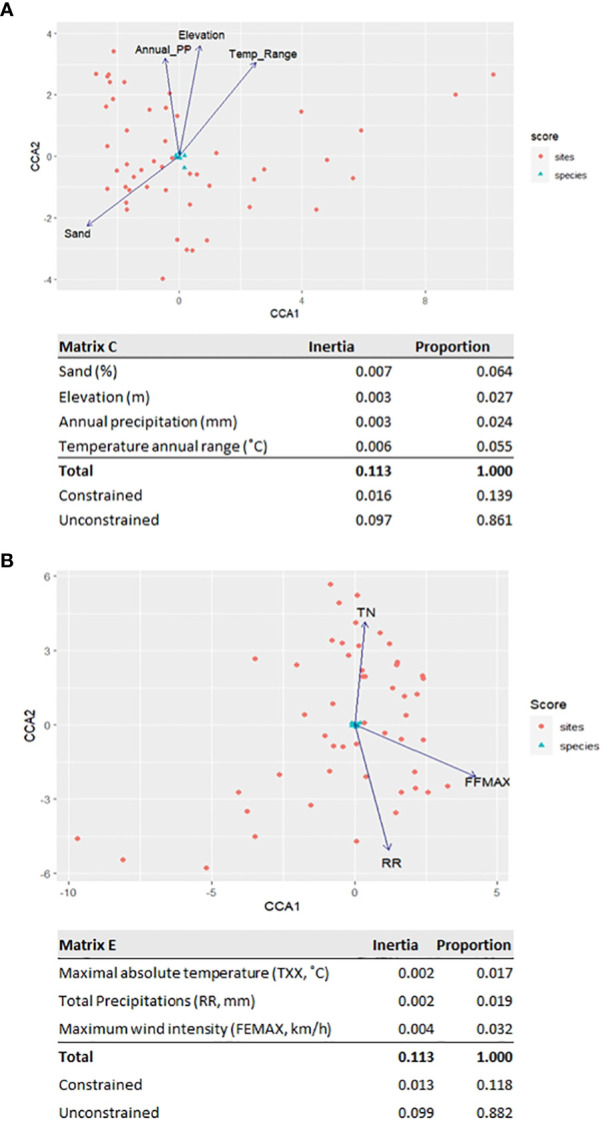
Canonical correspondence analysis showing the influence of the edaphoclimatic variables (arrows) in composition of the RAc profiles obtained for *Pinus pinaster* samples in the seven locations across the four sampling campaigns (numbers): **(A)** average of 30-year climatic data series and **(B)** climatic parameters measured in the month prior to the sampling.

A CCA was performed to scrutinize possible correlations between edaphoclimatic parameters and the observed diversity of resin acids in the *P. pinaster* rosin composite samples. First, the influence of an average of 30-year climatic data series and soil data was tested. By PCA, the parameters’ dependence was tested (**Supplementary Table 7**), and the complexity of matrices A and B was reduced, resulting in matrix D, which comprises four edaphoclimatic parameters (**Supplementary Table 8**) that retain >85% of the original data variance. The resultant CCA ordinations explain 11% of the variance of the RAc profiles (**Figure 6A**); the strongest correlation observed was the percentage of sand in the soil and the average annual temperature, which explains 6.4% and 5.5%, respectively.

Since the profile of resin acids in the rosin composite samples at a defined location showed some temporal variation (**Figure 5**), the available climatic parameters measured in the month prior to the sampling (IPMA, 2020)— matrix C—were used. PCA reduction generated matrix E, which comprises only three parameters (**Supplementary Table 9**), explaining 89% of the data variance. The resultant CCA ordination explains 8.8% of the variance of the RAc profiles (**Figure 6B**); the strongest correlation observed is with the maximum wind intensity, which explains 3.2% of the variability.

## 4 Discussion

*Pinus pinaster* oleoresin was collected from healthy resin-producing trees spread through seven different pure forest ecosystems within the continental territory of Portugal (**Figure 2**). The composite samples constitute a snapshot of the fingerprint of each forest at the moment of collection. These samples were subsequently distilled to obtain the corresponding rosin composite samples.

After some preliminary tests, NMR (and not GC-MS) was chosen as a gold standard method for the systematic analysis of all rosin samples, gathering their spectral profiles and quantifying the composing resin acids. The NMR data, either the spectral fingerprints (**Figure 3**) or the inferred quantifications of six resin acids (**Figures 1**, **4**), showed the similarity of all *P. pinaster* samples, which did not cluster per location. The oleoresin composition can differ between individuals from the same population due to genetic factors, among others (Arrabal et al., 2005; Arrabal et al., 2012; Kopaczyk et al., 2020; Luan et al., 2021), a variability herein unseen since strategically the oleoresin from 50 distinct *P. pinaster* trees was polled prior to distillation, similar to that performed in industry.

The observed chemical profiles of *P. pinaster* rosin, where the prevalent resin acid is AA, closely followed by DHA and IPA, are consistent with those reported previously for related samples collected within Portugal (Joye and Lawrence, 1967; Ghanmi et al., 2009; Simões et al., 2021; Alonso-Esteban et al., 2022; Rubini et al., 2022). Similar samples derived from the Corsica forest are rich in DHA and LEV instead (Ottavioli et al., 2019). The prevalence of AA observed in the oleoresin samples is biotechnologically relevant since several fungal strains can modify AA structure, yielding derivatives with interesting pharmacological activities (Özşen et al., 2017).

The PCA of *P. pinaster* samples shows a clear separation from *P. pinea* outlier samples mostly due to NEA, PA, and DHA abundances (**Figure 4**). This result is consistent with the idea that the rosin compositional profile is considered a chemotaxonomic indicator of pine species (Arrabal et al., 2012; Rubini et al., 2022). This means that the sampling strategy used allowed good discrimination between different pine species. Also, in phloem samples, the profiles are dominated by AA and DHA in *P. pinaster* and *P. pinea*, respectively (Simões et al., 2021).

To reduce the variability of the dataset with all collection sites and collection dates, the sampling dates were differentiated. This allowed us to observe that the RAc profiles suffered some alteration over time (**Figure 5**), suggesting that edaphoclimatic factors can impact oleoresin chemical composition. To test this hypothesis, canonical correspondence analyses resorting to available edaphoclimatic parameters from an average 30-year period or the month prior to the sampling were applied. The used edaphoclimatic data only explain ~11% of the observed resin acid diversity in *P. pinaster*. The sand percentage in soil and the average annual temperature (averaged analysis) and the maximum wind intensity (focused analysis) were those identified as to possibly influence the RAc profiles. These results are consistent with previous comparable CCA studies on pine forests, where the average annual temperature was suggested to influence the resin acid profile (Arrabal et al., 2005; Rajčević et al., 2019). The occurrence of cavitation due to strong winds has been suggested to influence oleoresin production (Ioannidis et al., 2019); however, the time-constrained CCA suggests a putative correlation of the wind intensity with the resin acid profiles as well. This observation deserves focused examination in the future. Additional variability apart from that described in both ordinations can be explained by parameters that are not represented in the generated matrices, including genotype (Rubini et al., 2022), pests (Gaspar et al., 2020; Gonçalves et al., 2020; Kopaczyk et al., 2020; López-Goldar et al., 2020), and proximity of forest fires (Cannac et al., 2009; Romero and Ganteaume, 2021), as suggested before. The impact of silvicultural conditions on resin composition, e.g., the resin tapping method, cannot be overlooked as well (Rubini et al., 2022). The climatic parameters here identified that influence, to a minor extent, the rosin composition are temperature and wind, possibly a consequence of their impact on the tree metabolism (biotic factors) and resin stability (abiotic factors).

In general, the chemical fingerprint of Portuguese rosin (specifically its RAc content) was observed to be rather stable regardless of location and collection time. This result shows that the quality of Portuguese rosin is very robust, an appealing characteristic of any industrial raw material. Moreover, their RAc profile is dominated by abietanes, more susceptible to chemical alteration due to the position of the conjugated double-bond system, which is absent in pimaranes (Silvestre and Gandini, 2008; Kugler et al., 2019). Finally, AA has adhesive, film-forming, and water-resistant properties; DHA has adhesive, corrosion resistance, and plasticizing properties; both are regarded as good candidates for the development of new drugs due to potential, e.g., anticancer, antimicrobial, and anti-inflammatory, properties. The abietanes are prone to further chemical syntheses; therefore, the Portuguese rosin can be seen as a starting point for the production of various end-products for different sectors, such as materials and pharma. Hence, it should inspire the development of innovative products/processes capable of boosting the competitiveness of the entire value chain, consequently leading to the implementation of stringent forest management measures that prevent decline due to fires and pest infestation and stimulate the planting of new forest areas.

## 5 Conclusion

In this study, an NMR method was used for a swift categorization of the diversity of resin acids extant in diverse rosin samples. The NMR results showed that the chemical fingerprint of Portuguese rosin (specifically their resin acid content) is mostly independent of the forest location, as well as the sampling period, possibly due to a weak correlation to edaphoclimatic parameters. More than half of their resin acid content consists of abietic acid and dehydroabietic acid. In conclusion, the stable chemistry and high abietane content of Portuguese *P. pinaster* rosin were herein validated: a regular high-performing raw industrial material is available across the entire continental territory of Portugal. This knowledge may therefore contribute to stimulating industry interest in oleoresin valorization, helping to reverse the progressive decline in the Portuguese resin tapping activity.

## Data availability statement

The datasets presented in this study can be found in online repositories. The names of the repository/repositories and accession number(s) can be found below: https://figshare.com/, https://doi.org/10.6084/m9.figshare.24119523.v1; https://figshare.com/, https://doi.org/10.6084/m9.figshare.24119691.v1.

## Author contributions

ÂP: Formal Analysis, Investigation, Writing – original draft. IM: Formal Analysis, Investigation, Writing – original draft. AB: Formal Analysis, Investigation, Writing – review & editing. RE: Investigation, Writing – review & editing. CN: Investigation, Writing – review & editing. AV: Investigation, Writing – review & editing. JN: Funding acquisition, Project administration, Resources, Supervision, Writing – review & editing. CAMA: Funding acquisition, Project administration, Resources, Supervision, Writing – review & editing. CSP: Funding acquisition, Project administration, Resources, Supervision, Conceptualization, Writing – review & editing.
