# Supplementary material for: Rosin from Pinus pinaster Portuguese forests shows a regular profile of resin acids
Source: Front Plant Sci. 2023 Oct 26;14:1268887. doi: 10.3389/fpls.2023.1268887 (PMC10640998; doi:10.3389/fpls.2023.1268887)
Supplement: Supplementary file 1 [file DataSheet_1.pdf]

Supplementary Material

Supplementary Figures

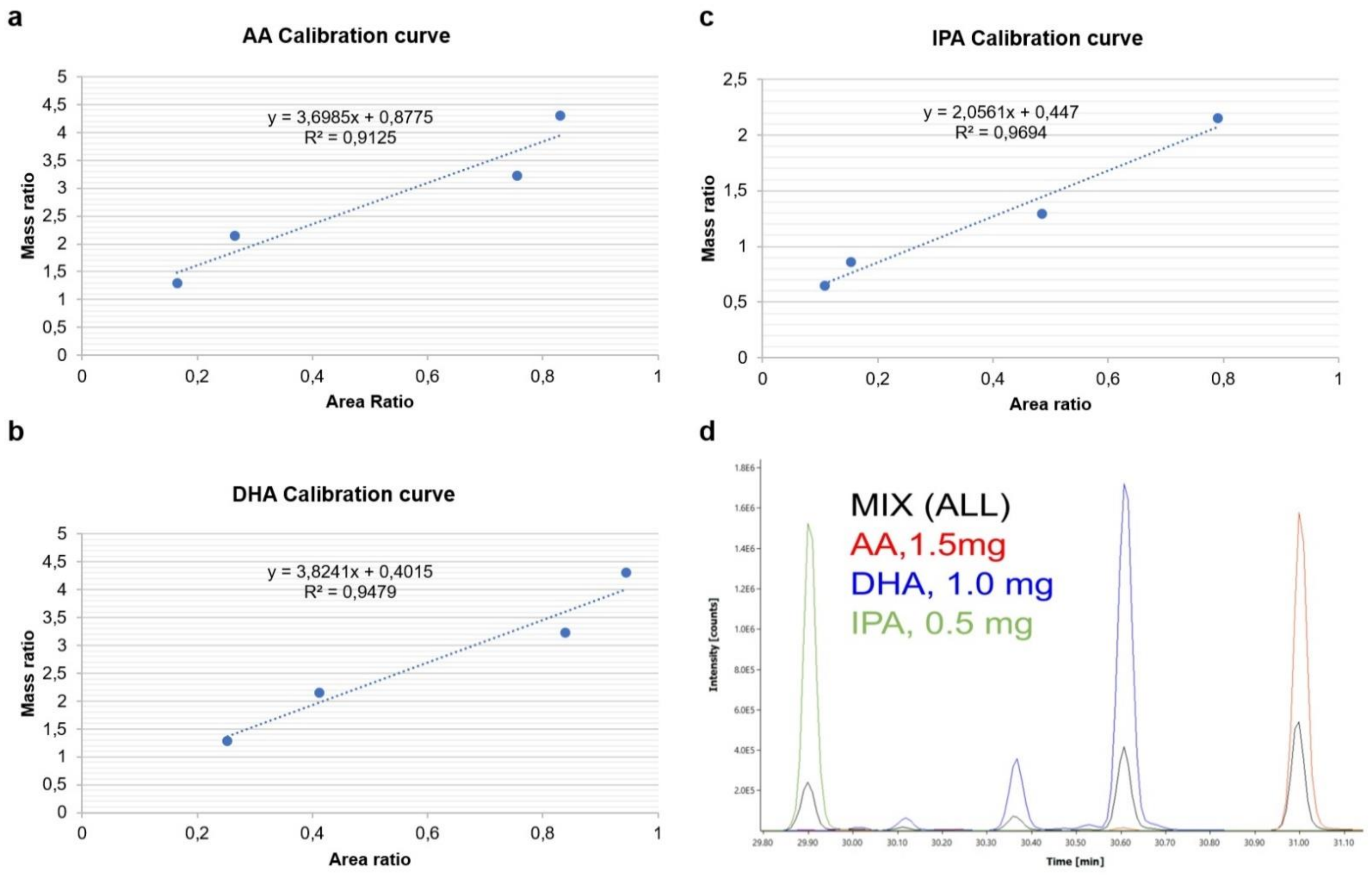

Supplementary Figure 1. GC-MS analysis of resin diterpenic acids standards: **a** – **c** calibration curves for abietic acid (AA, **a**), dehydroabietic acid (DHA, **b**) and isopimaric acid (IPA, **c**); **d** Overlay of Total Ion Chromatograms (TIC) showing a deviation in the quantification of AA, DHA and IPA analyzed as a mixture when compared to the individual analysis.

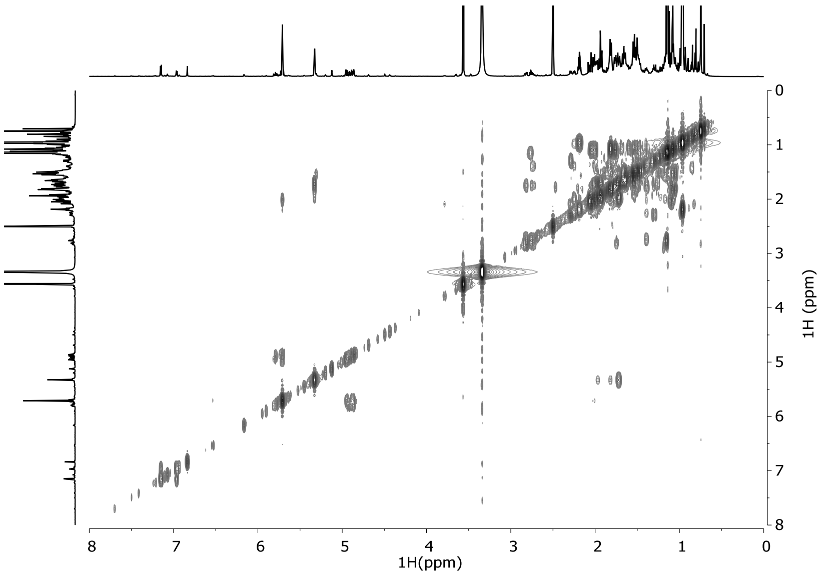

Supplementary Figure 2. 2D-^{1}H-^{1}H COSY-NMR spectrum (COrrelated SpectroscopY) of colophony sample from *Pinus pinaster* (1st collection date, 2018 from the location of P4) in DMSO-d_6.

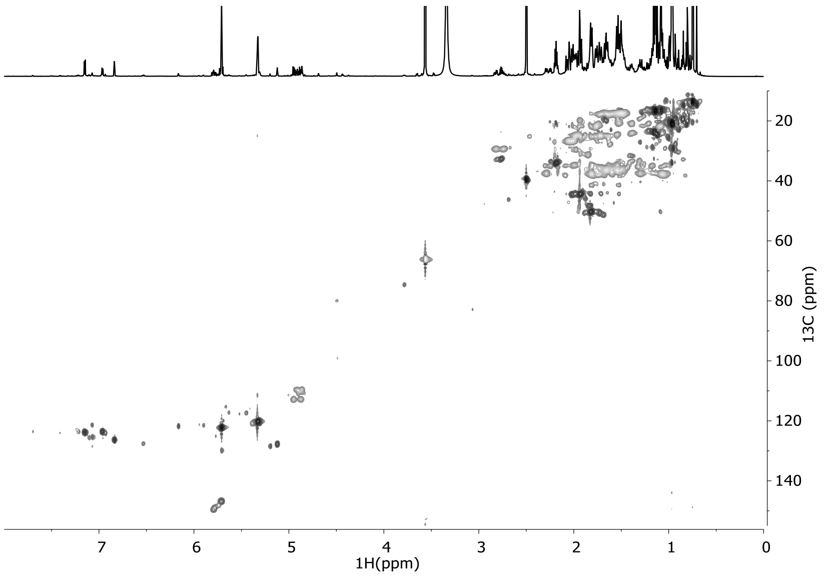

Supplementary Figure 3. 2D-^{1}H-^{13}C HSQC-NMR spectrum (Heteronuclear Single Quantum Coherence) of colophony sample from *Pinus pinaster* (1st collection date, 2018 from the location of P4) in DMSO-d_6.

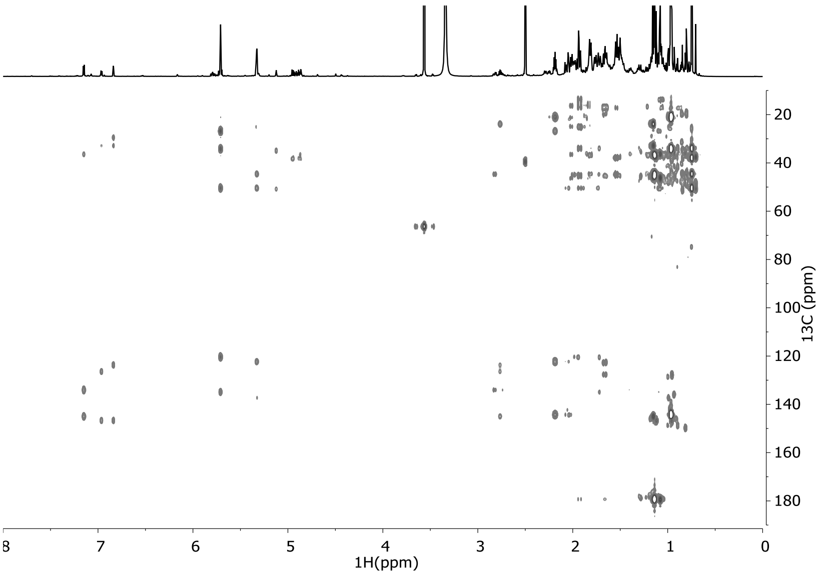

Supplementary Figure 4. 2D-^{1}H-^{13}C HMBC-NMR spectrum (Heteronuclear Multiple Bond Correlation) of colophony sample from *Pinus pinaster* (1st collection date, 2018 from the location of P4) in DMSO-d_6.

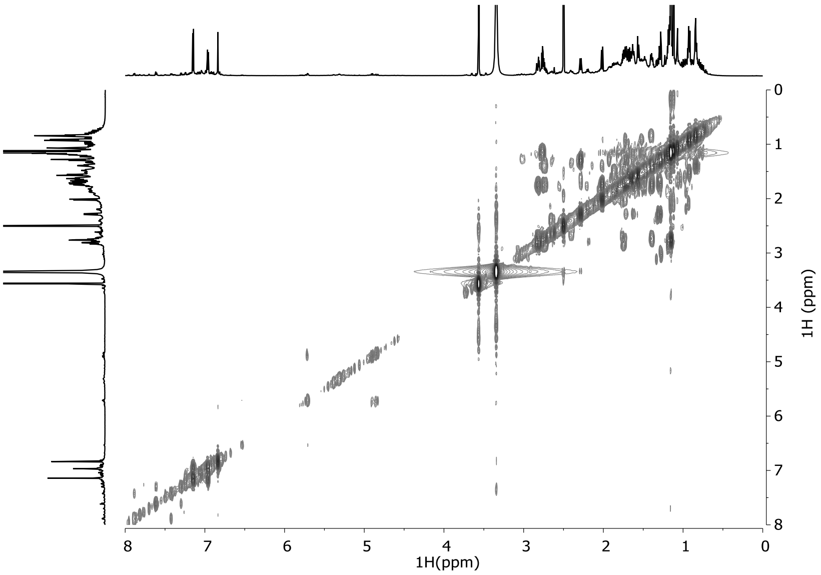

Supplementary Figure 5. 2D-^{1}H-^{1}H COSY-NMR spectrum (COrrelated SpectroscopY) of colophony sample from *Pinus pinea* (1st collection date, 2018 from the location of P8) in DMSO-d_6.

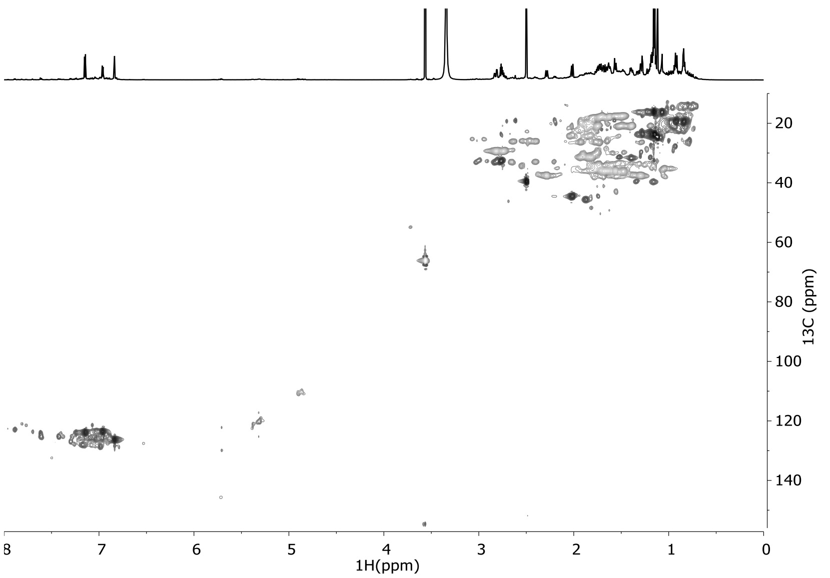

Supplementary Figure 6. 2D-^{1}H-^{13}C HSQC-NMR spectrum (Heteronuclear Single Quantum Coherence) of colophony sample from *Pinus pinea* (1st collection date, 2018 from the location of P8) in DMSO-d_6.

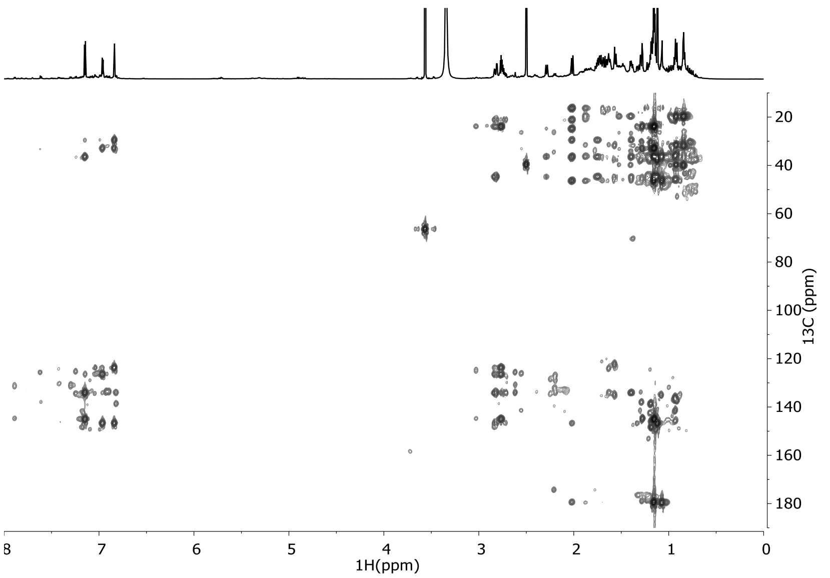

Supplementary Figure 7. 2D-^{1}H-^{13}C HMBC-NMR spectrum (Heteronuclear Multiple Bond Correlation) of colophony sample from *Pinus pinea* (1st collection date, 2018 from the location of P8) in DMSO-d_6.

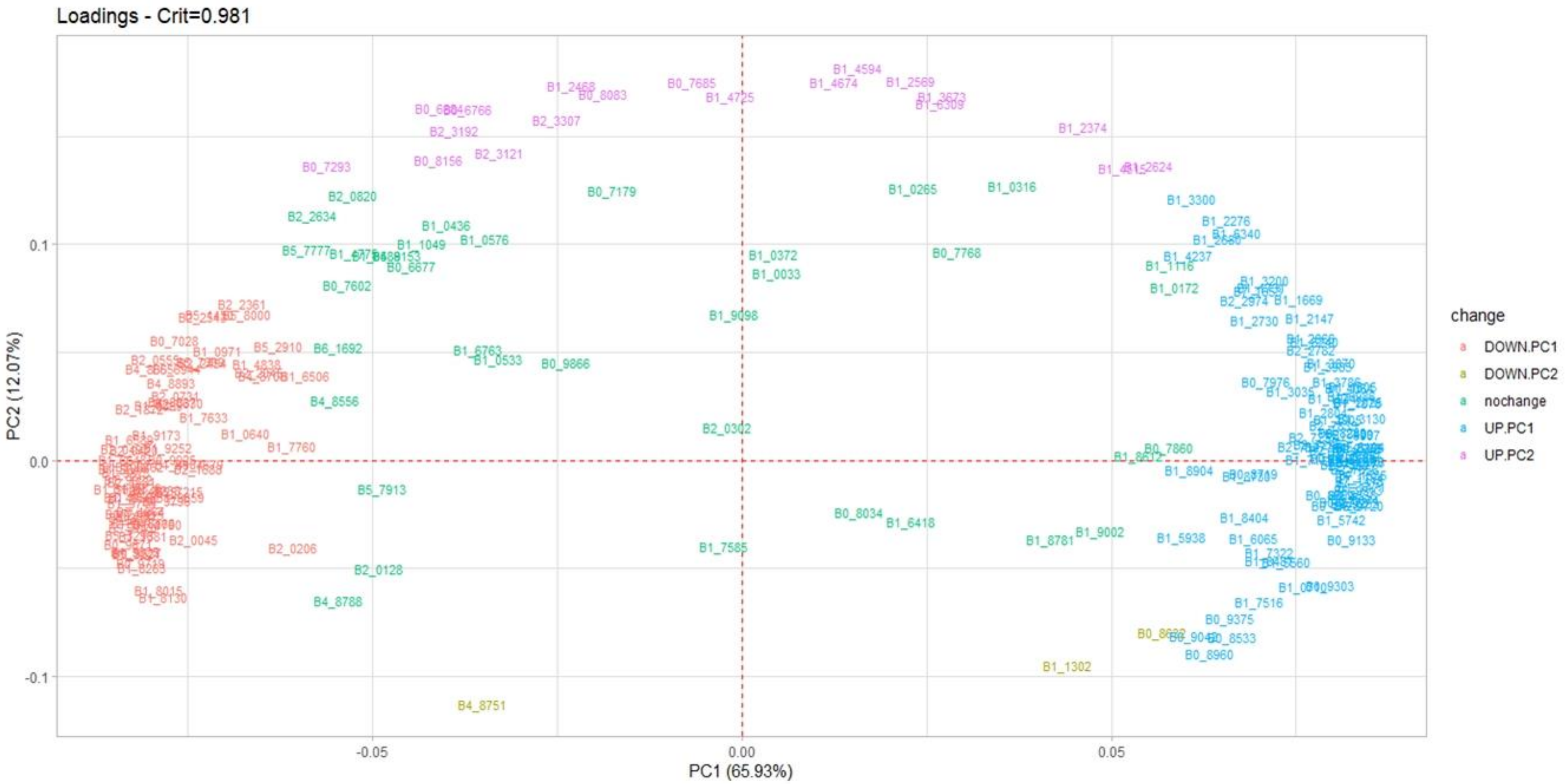

Supplementary Figure 8. PCA loadings obtained by statistical analysis of ^{1}H NMR spectra of the *Pinus* colophony samples collected in the continental Portuguese forestry.

^{1}H Chemical Shifts (ppm)	Resin acids
0.75	Abietic acid, AA
6.84	Dehydroabietic acid, DHA
6.16	Neoabietic acid, NEA
5.33	Abietic and Palustric acid, AA/ PAL
5.79	Isopimaric acid, IPA
5.12	Pimaric acid, PA

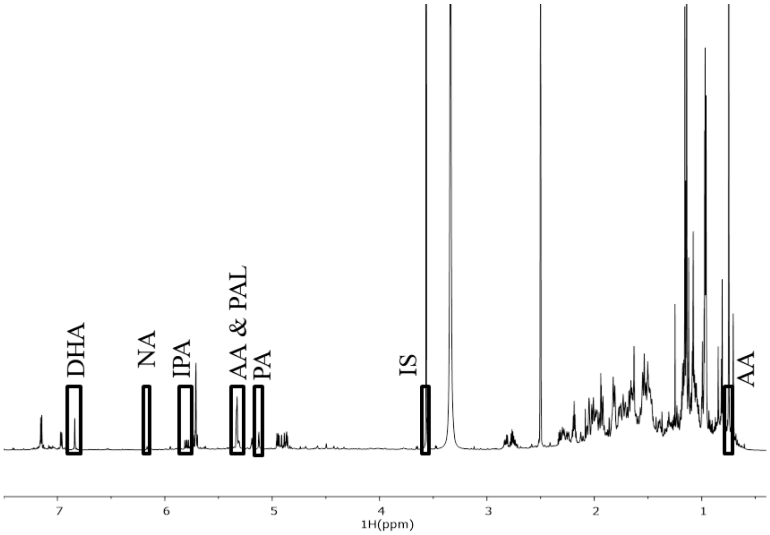

Supplementary Figure 9. ^{1}H Chemical shifts of resin acids used in colophony quantification.

Supplementary Tables

Supplementary Table 1. Dates of independent sampling campaigns on *Pinus pinaster* Portuguese forestry (Provenance codes P1-P7; P8 is a *Pinus pinea* forest)

				Sampling Campaigns (dates)			
Species	**P. code**	**Municipality**	**Location**	**1**	**2**	**3**	**4**
Pinus pinaster	P1	Paredes de Coura	Agualonga	8/8/2018	24/09/2018	10/10/2018	
	P2	Vila Pouca de Aguiar	Tresminas	2/8/2018	24/09/2018	10/10/2018	
	P3	Ourém	Caxarias	23/07/2018	18/09/2018	8/10/2018	14/09/2019
	P4	Covilhã	Tortosendo	16/08/2018	27/09/2018	24/10/2018	
	P5	Oleiros	Sarnadas S. Simão	16/08/2018	27/09/2018	24/10/2018	21/09/2019
	P6	Alcobaça	Pataias	17/08/2018	22/09/2018	24/11/2018	7/9/2019
	P7	Sines	Sines	23/08/2018	5/10/2018	17/11/2018	8/9/2019
P. pinea	P8	Alcochete	Alcohete			24/10/2018	20/02/2019

Supplementary Table 2. Climatic data from 30-year data series. Summary of 30-year climatic data series for *Pinus pinaster* sampling locations in continental Portugal (Matrix A, 21 climatic parameters, locations P1 – P7). Bioclimatic parameters of the seven studied locations were extracted from the WorldClim set of global climate layers (WorldClim 2020).

Province	Precipitation (mm)							
	per quarter				Seasonality	*per* month		Annual
	Colder	Warmer	Drier	Wetter	(Coefficient of Variation)	Drier	Wetter	
**P1**	637	153	130	637	50.566	30	229	1600
**P2**	489	94	89	489	53.548	16	170	1213
**P3**	344	59	55	347	55.378	10	122	851
**P4**	545	87	84	553	57.446	15	199	1315
**P5**	419	55	55	427	58.510	9	152	1020
**P6**	282	45	39	296	58.650	7	102	693
**P7**	262	30	24	279	66.604	3	103	600

Province	Temperature (°C)										
	Mean *per* quarter				Annual Range	Min and Max *per* month		Seasonality	Isothermality	Mean Diurnal Range	Annual Mean
	Colder	Warmer	Drier	Wetter	(BIO5-BIO6)	Colder	Warmer	(standard deviation ×100)	(BIO2/BIO7 x100)		
**P1**	7.803	18.453	18.375	7.803	20.548	3.941	24.488	437.134	41.433	8.513	12.916
**P2**	5.830	18.434	18.359	5.830	23.433	1.831	25.264	520.238	38.207	8.953	11.676
**P3**	9.976	20.980	20.687	10.766	22.105	5.453	27.558	447.106	42.886	9.480	15.233
**P4**	6.267	19.609	19.497	6.984	23.934	2.555	26.488	552.333	36.509	8.738	12.362
**P5**	8.292	21.785	21.785	8.966	25.094	4.166	29.260	556.838	38.320	9.616	14.611
**P6**	11.321	19.617	19.279	12.117	16.353	7.507	23.861	336.581	43.008	7.033	15.383
**P7**	11.305	20.730	20.481	12.158	20.155	7.174	27.329	385.315	45.598	9.190	15.938

BIO 2 = Mean Diurnal Range (Mean of monthly (max temp - min temp))

BIO5 = Max Temperature of Warmest Month

BIO 6 = Min Temperature of Coldest Month

BIO 7 = Temperature Annual Range (BIO5-BIO6)

Supplementary Table 3. Edaphic data for the *Pinus pinaster* sampling sites. A summary of soil properties for *Pinus pinaster* sampling locations in continental Portugal (Matrix B, 7 edaphic parameters, locations P1 – P7). Edaphic parameters of the seven studied locations were extracted from the INFOSOLO (INIAV 2016).

Province	Sand (%)	Silt (%)	Clay (%)	Elevation (m)	Sea Distance (km)
**P1**	82.9	7.8	9.3	415	30
**P2**	68.1	6.1	4.5	773	140
**P3**	83.3	3.6	13.1	166	60
**P4**	54.6	34.5	11,0	696	147
**P5**	32.1	41.8	26.1	584	132
**P6**	94.4	2.6	3,0	128	4
**P7**	97.4	1.6	1,0	53	6
**P8**	82.8	12.1	5,0	17	0.03

Supplementary Table 4. Climatic data from monthly series. A summary of climatic parameters measured in the month prior to the sampling at *Pinus pinaster* Portuguese forestry (Matrix C, 6 climatic parameters, locations P1 – P7). Bioclimatic parameters of the seven studied locations were extracted from the IPMA (IPMA 2020).

Code Region	TN (°C)	TX (°C)	TNN (°C)	TXX (°C)	RR (mm)	FFMAX (km/h)	Sampling Campaign
1	15.2	23.2	11.7	26.5	20.2	42.8	1
1	15.2	23.2	11.7	26.5	20.2	42.8	1
1	13.6	30.3	9.3	38.8	6.7	32	2
1	13.6	30.3	9.3	38.8	6.7	32	2
1	13.6	30.3	9.3	38.8	6.7	32	3
1	13.6	30.3	9.3	38.8	6.7	32	3
2	14.3	27	10.6	32.5	10.1	36	1
2	14.3	27	10.6	32.5	10.1	36	1
2	13.5	29.8	10	34.5	20.8	44.6	2
2	13.5	29.8	10	34.5	20.8	44.6	2
2	13.5	29.8	10	34.5	20.8	44.6	3
2	13.5	29.8	10	34.5	20.8	44.6	3
3	20	36	13.9	43.5	1	64.8	1
3	20	36	13.9	43.5	1	64.8	1
3	14.3	28.7	11.9	39.3	1	34.6	2
3	14.3	28.7	11.9	39.3	1	34.6	2
3	11.9	22.6	2.7	31.9	57	60.8	3
3	11.9	22.6	2.7	31.9	57	60.8	3
3	15.4	29.7	10.1	37.2	14.7	52.2	4
3	15.4	29.7	10.1	37.2	14.7	52.2	4
4	16	28	13.8	30.9	0	50.4	1
4	16	28	13.8	30.9	0	50.4	1
4	19.5	30.9	12.3	36.3	12.9	69.1	2
4	19.5	30.9	12.3	36.3	12.9	69.1	2
4	19.5	30.9	12.3	36.3	12.9	69.1	3
4	19.5	30.9	12.3	36.3	12.9	69.1	3
5	20	36	13.9	43.5	1	64.8	1
5	20	36	13.9	43.5	1	64.8	1
5	14.3	28.7	11.9	39.3	1	34.6	2
5	14.3	28.7	11.9	39.3	1	34.6	2
5	11.9	22.6	2.7	31.9	57	60.8	3
5	11.9	22.6	2.7	31.9	57	60.8	3
5	15.4	29.7	10.1	37.2	14.7	52.2	4
5	15.4	29.7	10.1	37.2	14.7	52.2	4
6	15.4	29	10.1	42.3	1.7	45	1
6	15.4	29	10.1	42.3	1.7	45	1
6	16.5	34.3	13.3	40.4	0.1	36.7	2
6	16.5	34.3	13.3	40.4	0.1	36.7	2
6	9.8	23.6	3.7	33.5	70.3	97.6	3
6	9.8	23.6	3.7	33.5	70.3	97.6	3
6	12.8	26.8	7.8	34.2	22.4	49	4
6	12.8	26.8	7.8	34.2	22.4	49	4
7	16.5	33.9	12.6	45.5	0	57.2	1
7	16.5	33.9	12.6	45.5	0	57.2	1
7	16.4	33.5	13	38.6	5.4	52.9	2
7	16.4	33.5	13	38.6	5.4	52.9	2
7	11	25.4	7.2	35	47.8	66.2	3
7	11	25.4	7.2	35	47.8	66.2	3
7	15	29.2	10.3	37.8	24.9	51.5	4
7	15	29.2	10.3	37.8	24.9	51.5	4

TN - Average of minimum temperature (Degrees Celsius)

TX - Maximum temperature average (Degrees Celsius)

TNN/D - Absolute minimum temperature (Degrees Celsius) and day of occurrence

TXX/D - Absolute maximum temperature (Degrees Celsius) and day of occurrence

RR - Total rainfall (millimeters)

FFMAX/D - Maximum wind intensity, gust (km/h) and day of occurrence

Supplementary Table 5. Quantitative GC-MS analysis of the standard resin acids (stRAc) analyzed as a mixture.

		Mix3[1]		Mix2[1]	
**stRAc**	**Measured mass (mg)**	**Estimated mass (mg)**	**% of deviation[2]**	**Estimated mass (mg)**	**% of deviation[2]**
AA	1.49	1.26 ± 0.023	-21%	0.89 ± 0.004	-67%
AA	0.50	0.42 ± 0.021	-20%	0.39 ± 0.0013	-30%
IPA	0.50	0.50 ± 0.02	-3%	0.40 ± 0.0013	-24%
IPA	0.15	0.16 ± 0.06	6%	0.16 ± 0.003	2%
DHA	1.00	1.05 ± 0.05	5%	-	
DHA	0.30	0.27 ± 0.013	-12%	-	

[1]Misture 3 contains AA, DHA and IPA; Mixture 2 contains AA and IPA

[2]The percentage of deviation indicated was calculated as the ratio between the stRAc masses quantified and the measured masses.

Supplementary Table 6. Chemical structures and ^{1}H and ^{13}C chemical shifts (ppm) of the resin acids identified in the colophony samples. Signals not resolved in the NMR spectra due to overlapping signals or not yet assigned are not shown in this table.

Chemical structure	Chemical shifts (ppm)
**Resin acids**	
**ABIETIC ACID (AA)** 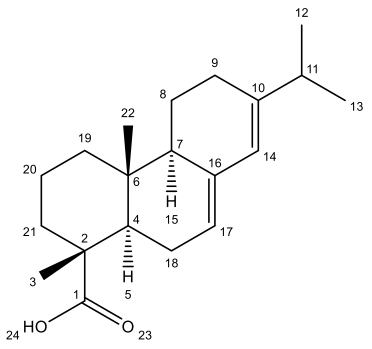	**^{1}H NMR** (800.33 MHz, DMSO-d_6) δ 0.78 (m, 3H, H-22), 0.82 (m, 6H, H-13, 12), 1.18 (m, 3H, H-3), 1.50 (m, 2H, H-20), 1.59 (m, 1H, H-19), 1.69 (m, 1H, H-19’), 1.73 (m, 2H, H-18), 1.77 (m, 2H, H-8), 1.78 (m, 2H, H-21), 2.00 (m, 2H, H-9), 2.18 (m, 1H, H-11), 5.33 (m, 1H, H-17), 5.72 (m, 1H, H-14).<br><br>**^{13}C NMR** (201.42 MHz, DMSO-d_6) δ 13.17 (C-22), 15.90 (C-3), 17.14 (C-20), 19.07 (C-13; 12),<br><br>20.57 (C-8), 24.41 (C-18), 26.07 (C-9), 33.19 (C-11), 35.96 (C-19), 37.49 (C-21); 120.29 (C-17), 121.92 (C-14).
**DEHYDROABIETIC ACID (DHA)** 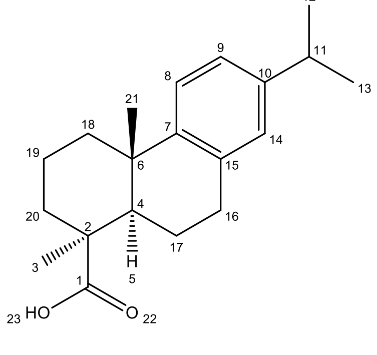	**^{1}H NMR** (800.33 MHz, DMSO-d_6) δ 1.17 (m, 6H, H-12, 13), 2.78 (m, 1H, H-11), 6.85 (s, 1H, H-14), 6.97 (m, 1H, H-9), 7.14 (m, 1H, H-8).<br><br>**^{13}C NMR** (201.42 MHz, DMSO-d_6) δ 23.31 (C-12, 13), 32.31 (C-11), 123.12 (C-9), 123.34 (C-8), 125.90 (C-14).

NEOABIETIC ACID (NEA)

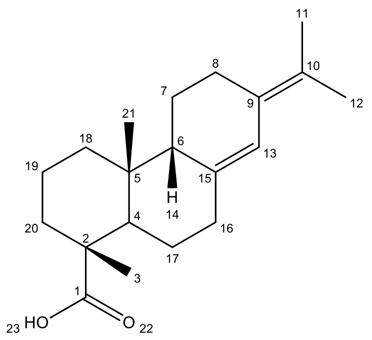

^{1}H NMR (800.33 MHz, DMSO-d_6) δ 1.67 (m, 1H, H-11-12), 6.16 (s, 1H, H-13).

^{13}C NMR (201.42 MHz, DMSO-d_6) δ 19.65 (C-11-12), 121.65 (C-13).

PALUSTRIC ACID (PAL)

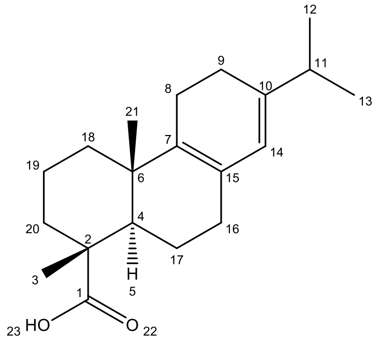

^{1}H NMR (800.33 MHz, DMSO-d_6) δ 5.33 (s, 1H, H-14).

^{13}C NMR (201.42 MHz, DMSO-d_6) δ 120.11 (C-14).

ISOPIMARIC ACID (IPA)

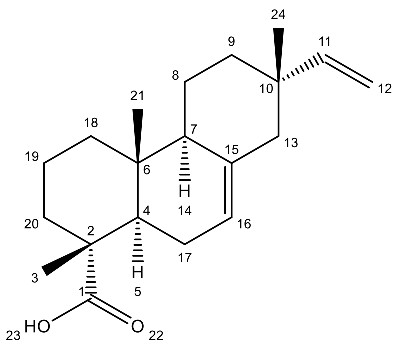

^{1}H NMR (800.33 MHz, DMSO-d_6) δ 1.92 (m, 1H, H-17), 4.87 (m, 1H, H-12), 4.93 (m, 1H, H-12’), 5.22 (s, 1H, H-16), 5.81 (m, 1H, H-11).

^{13}C NMR (201.42 MHz, DMSO-d_6) δ 30.63 (C-17), 109.06 (C-12), 124.70 (C-16), 149.36 (C-11).

**PIMARIC ACID (PA)**	**^{1}H NMR** (800.33 MHz, DMSO-d_6) δ 4.88 (m, 1H, H-12), 4.94 (m, 1H, H-12’), 5.14 (s, 1H, H-13), 5.73 (m, 1H, H-11).
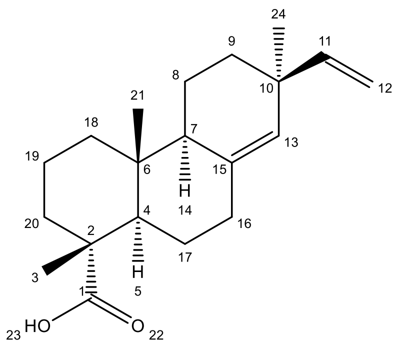	**^{13}C NMR** (201.42 MHz, DMSO-d_6) δ 112.09 (C-12), 127.50 (C-13), 146.48 (C-11).

Supplementary Table 7. Selection of significant axes from principal component analyses (PCA) of climate and soil variables (Matrix A to C) across the *Pinus pinaster* sampling locations by the Pearson's method (retaining >80% of the variability, left panel) and from reduced matrices (right panel).

Matrix_A	PC1	PC2	PC3	PC4	PC5	PC6
Precipitation of Coldest Quarter (mm)	-0.271	-0.015	-0.19	0.1475	-0.14	-0.217
Precipitation of Warmest Quarter (mm)	-0.255	0.1137	-0.305	-0.039	0.0063	0.3805
Precipitation of Driest Quarter (mm)	-0.268	0.0754	-0.217	-0.033	0.0745	0.0787
Precipitation of Wettest Quarter (mm)	-0.269	-0.017	-0.197	0.1832	-0.192	-0.169
Precipitation Seasonality (Coefficient of Variation) (mm)	0.2363	-0.104	0.0655	0.1044	-0.877	0.3002
Precipitation of Driest Month (mm)	-0.248	0.1185	-0.354	0.0085	0.0581	0.4553
Precipitation of Wettest Month (mm)	-0.265	-0.023	-0.227	0.1886	-0.267	-0.343
Annual Precipitation (mm)	-0.272	-1E-04	-0.194	0.1269	-0.057	-0.163
Mean Temperature of Coldest Quarter (°C)	0.2592	0.1265	-0.245	0.0728	0.0467	0.0868
Mean Temperature of Warmest Quarter (°C)	0.2085	-0.285	-0.211	0.2835	0.1692	0.1136
Mean Temperature of Driest Quarter (°C)	0.194	-0.312	-0.215	0.2944	0.1622	0.1013
Mean Temperature of Wettest Quarter (°C)	0.265	0.1004	-0.206	0.1203	0.0399	-0.448
Temperature Annual Range (°C)	-0.123	-0.432	0.0373	-0.123	0.0175	0.0156
Min Temperature of Coldest Month (°C)	0.2541	0.1587	-0.219	0.1566	-0.038	-0.088
Max Temperature of Warmest Month (°C)	0.1181	-0.426	-0.198	0.0063	-0.018	-0.078
Temperature Seasonality (standard deviation x100) (°C)	-0.169	-0.373	0.1572	0.1277	0.0462	-0.109
Isothermality (°C)	0.2074	0.2149	-0.339	-0.49	-0.123	-0.233
Mean Diurnal Range[1] (°C)	0.0031	-0.415	-0.267	-0.615	-0.053	-0.022
Annual Mean Temperature (°C)	0.2658	-0.001	-0.272	0.1329	0.0944	0.139

Matrix_B	PC1	PC2	PC3	PC4	PC5
Sand (%)	-0.498	0.1152	0.072	-0.078	-0.853
Silt (%)	0.4546	-0.319	-0.717	-0.238	-0.347
Clay (%)	0.408	-0.582	0.6533	-0.032	-0.259
Elevation (km)	0.4111	0.6177	0.2317	-0.624	-0.08
Sea Distance (km)	0.4585	0.4056	0.0004	0.7395	-0.28

Matrix_C	PC1	PC2	PC3	PC4	PC5	PC6
TN. Mean Min Temperature (°C)	-0.424	-0.464	0.2812	0.7093	-0.037	0.1486
TX. Mean Max Temperature (°C)	-0.468	0.204	0.2149	-0.232	-0.777	-0.189
TNN. Absolute Min Temperature (°C)	-0.465	-0.38	-0.08	-0.587	0.2128	0.4937
TXX. Absolute Max Temperature (°C)	-0.368	0.7462	0.2702	0.1278	0.3919	0.2547
RR. Precipitation (mm)	0.471	0.0638	0.2953	0.0317	-0.374	0.7391
FFMAX. Maximum wind intensity (km/h)	0.1695	-0.195	0.8415	-0.286	0.2364	-0.296

Matrix_A	PC1	PC2	PC3	PC4	PC5	PC6	PC7	PC8
Standard deviation	3.6792	2.0727	1.09439	0.76925	0.59605	0.13954	0.05913	1.17E-15
Proportion of Variance	0.6768	0.2148	0.05988	0.02959	0.01776	0.00097	0.00017	0.00E+00
Cumulative Proportion	0.6768	0.8916	0.9515	0.98109	0.99885	0.99983	1	1.00E+00

Matrix_B	PC1	PC2	PC3	PC4	PC5
Standard deviation	1.986	0.8741	0.44226	0.28399	0.12481
Proportion of Variance	0.7888	0.1528	0.03912	0.01613	0.00312
Cumulative Proportion	0.7888	0.9416	0.98075	0.99688	1

Matrix_C	PC1	PC2	PC3	PC4	PC5	PC6
Standard deviation	1.9775	1.0926	0.8107	0.35173	0.30173	0.15444
Proportion of Variance	0.6517	0.1989	0.1095	0.02062	0.01517	0.00398
Cumulative Proportion	0.6517	0.8507	0.9602	0.98085	0.99602	1

Supplementary Table 8. Reduced edaphoclimatic data from 30-year climatic data series (Matrix A) and soil properties (Matrix B) on *Pinus pinaster* sampling sites. A summary of the selected edaphoclimatic variables by PCA for further Canonical Correspondence Analysis (CCA) (Matrix D, 4 edaphoclimatic parameters, locations P1 – P7).

Code Region	Sand % (w/w)	Elevation (m)	Annual Precipitation (mm)	Temperature Annual Range (°C)
P1_1	82.9	415	1600	20.55
P1_2	82.9	415	1600	20.55
P1_3	82.9	415	1600	20.55
P1_4	82.9	415	1600	20.55
P1_5	82.9	415	1600	20.55
P1_6	82.9	415	1600	20.55
P2_1	68.1	773	1213	23.43
P2_2	68.1	773	1213	23.43
P2_3	68.1	773	1213	23.43
P2_4	68.1	773	1213	23.43
P2_5	68.1	773	1213	23.43
P2_6	68.1	773	1213	23.43
P3_1	83.3	166	851	22.1
P3_2	83.3	166	851	22.1
P3_3	83.3	166	851	22.1
P3_4	83.3	166	851	22.1
P3_5	83.3	166	851	22.1
P3_6	83.3	166	851	22.1
P3_7	83.3	166	851	22.1
P3_8	83.3	166	851	22.1
P4_1	54.6	696	1315	23.93
P4_2	54.6	696	1315	23.93
P4_3	54.6	696	1315	23.93
P4_4	54.6	696	1315	23.93
P4_5	54.6	696	1315	23.93
P4_6	54.6	696	1315	23.93
P5_1	32.1	584	1020	25.09
P5_2	32.1	584	1020	25.09
P5_3	32.1	584	1020	25.09
P5_4	32.1	584	1020	25.09
P5_5	32.1	584	1020	25.09
P5_6	32.1	584	1020	25.09
P5_7	32.1	584	1020	25.09
P5_8	32.1	584	1020	25.09
P6_1	94.4	128	693	16.35
P6_2	94.4	128	693	16.35
P6_3	94.4	128	693	16.35
P6_4	94.4	128	693	16.35
P6_5	94.4	128	693	16.35
P6_6	94.4	128	693	16.35
P6_7	94.4	128	693	16.35
P6_8	94.4	128	693	16.35
P7_1	97.4	53	600	20.16
P7_2	97.4	53	600	20.16
P7_3	97.4	53	600	20.16
P7_4	97.4	53	600	20.16
P7_5	97.4	53	600	20.16
P7_6	97.4	53	600	20.16
P7_7	97.4	53	600	20.16
P7_8	97.4	53	600	20.16

Supplementary Table 9. Reduced climatic data from monthly series. A summary of the selected monthly series climatic variables by PCA (from Matrix C) for further Canonical Correspondence Analysis (CCA) (Matrix E, 3 climatic parameters, locations P1 – P7).

Code Region	TN (°C)	RR (mm)	FFMAX (km/h)	Sampling Campaign
1	15.2	20.2	42.8	1
1	15.2	20.2	42.8	1
1	13.6	6.7	32	2
1	13.6	6.7	32	2
1	13.6	6.7	32	3
1	13.6	6.7	32	3
2	14.3	10.1	36	1
2	14.3	10.1	36	1
2	13.5	20.8	44.6	2
2	13.5	20.8	44.6	2
2	13.5	20.8	44.6	3
2	13.5	20.8	44.6	3
3	20	1	64.8	1
3	20	1	64.8	1
3	14.3	1	34.6	2
3	14.3	1	34.6	2
3	11.9	57	60.8	3
3	11.9	57	60.8	3
3	15.4	14.7	52.2	4
3	15.4	14.7	52.2	4
4	16	0	50.4	1
4	16	0	50.4	1
4	19.5	12.9	69.1	2
4	19.5	12.9	69.1	2
4	19.5	12.9	69.1	3
4	19.5	12.9	69.1	3
5	20	1	64.8	1
5	20	1	64.8	1
5	14.3	1	34.6	2
5	14.3	1	34.6	2
5	11.9	57	60.8	3
5	11.9	57	60.8	3
5	15.4	14.7	52.2	4
5	15.4	14.7	52.2	4
6	15.4	1.7	45	1
6	15.4	1.7	45	1
6	16.5	0.1	36.7	2
6	16.5	0.1	36.7	2
6	9.8	70.3	97.6	3
6	9.8	70.3	97.6	3
6	12.8	22.4	49	4
6	12.8	22.4	49	4
7	16.5	0	57.2	1
7	16.5	0	57.2	1
7	16.4	5.4	52.9	2
7	16.4	5.4	52.9	2
7	11	47.8	66.2	3
7	11	47.8	66.2	3
7	15	24.9	51.5	4
7	15	24.9	51.5	4

TN - Average of minimum temperature (Degrees Celsius)

RR - Total rainfall (millimeters)

FFMAX/D - Maximum wind intensity, gust (km/h) and day of occurrence
